# A cohort study of forced vital capacity, airway obstruction, and survival in the multinational Burden of Obstructive Lung Disease study

**DOI:** 10.1093/ije/dyag093

**Published:** 2026-06-07

**Authors:** Peter G J Burney, Emmanouil Bagkeris, James Potts, Rune Nielsen, Graham Devereux, Karima El Rhazi, Althea Aquart-Stewart, Asaad A Nafees, Parvaiz Koul, Padukudru Anand Mahesh, Dhiraj Agarwal, Sanjay Juvekar, Thorarinn Gislason, Meriam Denguezli, Imed Harrabi, Stefanni Nonna M Paraguas, Rain Jogi, Gregory Erhabor, A Sonia Buist, Christer Janson, Andre F S Amaral, BOLD Collaborative Research Group, Hasan Hafizi, Anila Aliko, Donika Bardhi, Holta Tafa, Natasha Thanasi, Arian Mezini, Alma Teferici, Dafina Todri, Jolanda Nikolla, Rezarta Kazasi, Hamid Hacene Cherkaski, Amira Bengrait, Tabarek Haddad, Ibtissem Zgaoula, Maamar Ghit, Abdelhamid Roubhia, Soumaya Boudra, Feryal Atoui, Randa Yakoubi, Rachid Benali, Abdelghani Bencheikh, Nadia Ait-Khaled, Christine Jenkins, Guy Marks, Tessa Bird, Paola Espinel, Kate Hardaker, Brett Toelle, Michael Studnicka, Torkil Dawes, Bernd Lamprecht, Lea Schirhofer, Herve Lawin, Arsene Kpangon, Karl Kpossou, Gildas Agodokpessi, Paul Ayelo, Benjamin Fayomi, Rolus Atrokpo, Gaston Hounton, Dieudonnè Yadjodo, Bertrand Mbatchou, Atongno Humphrey Ashu, Wan C Tan, Wen Wang, NanShan Zhong, Shengming Liu, Jiachun Lu, Pixin Ran, Dali Wang, Jin-ping Zheng, Yumin Zhou, Rain Jõgi, Hendrik Laja, Katrin Ulst, Vappu Zobel, Toomas-Julius Lill, Katrin Kiili, Ira Laanelepp, Tobias Welte, Isabelle Bodemann, Henning Geldmacher, Alexandra Schweda-Linow, Thorarinn Gislason, Bryndis Benedikdtsdottir, Kristin Jörundsdottir, Lovisa Gudmundsdottir, Sigrun Gudmundsdottir, Gunnar Gudmundsson, Elin Helga Thorarinsdottir, Hjördis Sigrun Pálsdottir, Padukudru Mahesh Anand, Parvaiz A Koul, Sajjad Malik, Nissar A Hakim, Umar Hafiz Khan, Rohini Chowgule, Vasant Shetye, Jonelle Raphael, Rosel Almeda, Tawde Mahesh, Rafiq Tadvi, Sunil Katkar, Milind Kadam, Rupesh Dhanawade, Umesh Ghurup, Sanjay Juvekar, Siddhi Hirve, Somnath Sambhudas, Bharat Chaidhary, Meera Tambe, Savita Pingale, Arati Umap, Archana Umap, Nitin Shelar, Sampada Devchakke, Sharda Chaudhary, Suvarna Bondre, Savita Walke, Ashleshsa Gawhane, Anil Sapkal, Rupali Argade, Vijay Gaikwad, Dhiraj Agrawal, Babu Pawar, Shalan Mhetre, Namdev Kale, Shirish Kathale, Sundeep Salvi, Bill Brashier, Jyoti Londhe, Sapna Madas, Althea Aquart-Stewart, Akosua Francia Aikman, Talant M Sooronbaev, Bermet M Estebesova, Meerim Akmatalieva, Saadat Usenbaeva, Jypara Kydyrova, Eliza Bostonova, Ulan Sheraliev, Nuridin Marajapov, Nurgul Toktogulova, Berik Emilov, Toktogul Azilova, Gulnara Beishekeeva, Nasyikat Dononbaeva, Aijamal Tabyshova, Kevin Mortimer, Wezzie Nyapigoti, Ernest Mwangoka, Mayamiko Kambwili, Martha Chipeta, Gloria Banda, Suzgo Mkandawire, Justice Banda, Graham Devereux, Jamie Rylance, Martin Njoroge, Catherine Chirwa, Chifundo Mhango, Edgar Ngwira, Faith Zumazuma, Frank Jonas, Patrick Mjojo, Li-Cher Loh, Abdul Rashid, Siti Sholehah, Mohamed C Benjelloun, Chakib Nejjari, Mohamed Elbiaze, Karima El Rhazi, Manelle Rjimati, Btissame ElHarche, Reda Benjelloun, Yassin Chefchaou, E F M Wouters, G J Wesseling, Daniel Obaseki, Gregory Erhabor, Olayemi Awopeju, Olufemi Adewole, Amund Gulsvik, Tina Endresen, Lene Svendsen, Rune Nielsen, Marit Aardal, Hildegunn B Fleten, Gerd Eli Dale, Eli Nordeide, Malin P Grøttveit, Åsa Skjelde, Ane Aamli Gagnat, Anders Ørskov Rotevatn, Marta Erdal, Asaad A Nafees, Muhammad Irfan, Hasan Nawaz Tahir, Muhammad Noman, Roman Ul Haq, Luisito F Idolor, Teresita S de Guia, Norberto A Francisco, Camilo C Roa, Fernando G Ayuyao, Cecil Z Tady, Daniel T Tan, Sylvia Banal-Yang, Vincent M Balanag, Maria Teresita N Reyes, Renato B Dantes, Stefanni Nonna M Paraguas, Renato B Dantes, Lourdes Amarillo, Lakan U Berratio, Lenora C Fernandez, Norberto A Francisco, Gerard S Garcia, Teresita S de Guia, Luisito F Idolor, Sullian S Naval, Thessa Reyes, Camilo C Roa, Ma Flordeliza Sanchez, Leander P Simpao, Ewa Nizankowska-Mogilnicka, Jakub Frey, Rafal Harat, Filip Mejza, Pawel Nastalek, Andrzej Pajak, Wojciech Skucha, Andrzej Szczeklik, Magda Twardowska, Cristina Bárbara, Fátima Rodrigues, Hermínia Dias, João Cardoso, João Almeida, Maria João Matos, Paula Simão, Moutinho Santos, Reis Ferreira, M Al Ghobain, H Alorainy, E El-Hamad, M Al Hajjaj, A Hashi, R Dela, R Fanuncio, E Doloriel, I Marciano, L Safia, Eric Bateman, Anamika Jithoo, Desiree Adams, Edward Barnes, Jasper Freeman, Anton Hayes, Sipho Hlengwa, Christine Johannisen, Mariana Koopman, Innocentia Louw, Ina Ludick, Alta Olckers, Johanna Ryck, Janita Storbeck, Richard van Zyl-Smit, Kirthi Gunasekera, Rajitha Wickremasinghe, Asma Elsony, Hana A Elsadig, Nada Bakery Osman, Bandar Salah Noory, Monjda Awad Mohamed, Hasab Alrasoul Akasha Ahmed Osman, Namarig Moham ed Elhassan, Abdel Mu‘is El Zain, Marwa Mohamed Mohamaden, Suhaiba Khalifa, Mahmoud Elhadi, Mohand Hassan, Dalia Abdelmonam, Rana Ahmed, Rashid Osman, Hind Eltigani, Najlaa Mohamed Abass, Ahmed Beriar Ahmed, Sahar AlaElddin, Christer Janson, Inga Sif Olafsdottir, Katarina Nisser, Ulrike Spetz-Nyström, Gunilla Hägg, Gun-Marie Lund, Andrei Malinovschi, Eva Wallberg, Birgitta Appelfeldt, Mona Andrén, Terence Seemungal, Fallon Lutchmansingh, Liane Conyette, Imed Harrabi, Myriam Denguezli, Zouhair Tabka, Hager Daldoul, Zaki Boukheroufa, Firas Chouikha, Wahbi Belhaj Khalifa, Safa Hsan, Nadia Lakhdar, Mounir Landolsi, Ali Kocabaş, Attila Hancioglu, Ismail Hanta, Sedat Kuleci, Ahmet Sinan Turkyilmaz, Sema Umut, Turgay Unalan, Peter G J Burney, Anamika Jithoo, Louisa Gnatiuc, Hadia Azar, Jaymini Patel, Caron Amor, James Potts, Michael Tumilty, Fiona McLean, Risha Dudhaiya, Andre F S Amaral, Octavia Mulhern, Emmanouil Bagkeris, Jasleen Gegic, Paul Cullinan, Cosetta Minelli, A Sonia Buist, Mary Ann McBurnie, William M Vollmer, Suzanne Gillespie, Sean Sullivan, Todd A Lee, Kevin B Weiss, Robert L Jensen, Robert Crapo, Paul Enright, David M Mannino, John Cain, Rebecca Copeland, Dana Hazen, Jennifer Methvin, Vanessa Garcia Larsen

**Affiliations:** National Heart and Lung Institute, Imperial College London, London, United Kingdom; National Heart and Lung Institute, Imperial College London, London, United Kingdom; National Heart and Lung Institute, Imperial College London, London, United Kingdom; Department of Clinical Science, University of Bergen, Bergen, Norway; Department of Thoracic Medicine, Haukeland University Hospital, Bergen, Norway; Department of Clinical Sciences, Liverpool School of Tropical Medicine, Liverpool, United Kingdom; Department of Epidemiology and Public Health, Faculty of Medicine, Dentistry and Pharmacy, Sidi Mohamed Ben Abdillah University, Hassan II University Hospital Center of Fez, Fez, Morocco; Department of Medicine, University of the West Indies, Mona Campus, Kingston, Jamaica; Department of Community Health Sciences, Aga Khan University, Karachi, Pakistan; Department of Pulmonary Medicine, Sheri Kashmir Institute of Medical Sciences, Srinagar, India; Department of Respiratory Medicine, JSS Medical College, Mysuru, Karnataka, India; Vadu Rural Health Program, KEM Hospital Research Centre, Pune, India; Vadu Rural Health Program, KEM Hospital Research Centre, Pune, India; Dr D.Y. Patil Medical College, Hospital and Research Centre, Dr D.Y. Patil Vidyapeeth, Pimpri, Pune, India; Faculty of Medicine, University of Iceland, Reykjavik, Iceland; Department of Sleep, Landspitali University Hospital, Reykjavik, Iceland; Faculté de Médecine Dentaire de Monastir, Université de Monastir, Monastir, Tunisia; Ibn El Jazzar Faculty of Medicine of Sousse, University of Sousse, Sousse, Tunisia; Philippine College of Chest Physicians, Quezon City, Philippines; Philippine Heart Centre, Quezon City, Philippines; Lung Clinic, Tartu University Hospital, Tartu, Estonia; Department of Medicine, Obafemi Awolowo University/Obafemi Awolowo University Teaching Hospital Complex, Ile-Ife, Osun State, Nigeria; School of Medicine, Oregon Health and Science University, Portland, OR, United States; Department of Medical Sciences: Respiratory, Allergy and Sleep Research, Uppsala University, Uppsala, Sweden; National Heart and Lung Institute, Imperial College London, London, United Kingdom; NIHR Imperial Biomedical Research Centre, London, United Kingdom

**Keywords:** survival analysis, vital capacity, airflow obstruction, multinational, lung function

## Abstract

**Background:**

In the USA, higher forced vital capacity (FVC) is linked with longer survival, and FVC is associated with survival independently of ethnicity. The implications for the low FVC values in parts of Asia and Africa are unknown.

**Methods:**

We used data from 16 sites of the multinational Burden of Obstructive Lung Disease (BOLD) study that completed follow-up of participants between 2019 and 2021 and reported at least five deaths between baseline and follow-up. We assessed the association of mortality with FVC and Forced Expiratory Volume in 1 second (FEV_1_)/FVC ratio within each site using Cox proportional hazards models. These models were adjusted for age, smoking, height, and weight. Effect estimates from all sites were combined using meta-analysis. Systematic regional differences were investigated.

**Results:**

Of 9927 study participants with follow-up data, 1120 (11.3%) had died [mean follow-up = 8.7 years, standard deviation (SD) = 3.3 years]. Baseline post-bronchodilator FVC and FEV_1_/FVC were inversely associated with mortality. When both FVC and FEV_1_/FVC were mutually adjusted for each other, the decreased mortality rates were more pronounced for each SD higher FVC at baseline [44% (95% confidence interval (CI): 25%, 58%) for men and 28% (95% CI: 11%, 41%) for women] than for FEV_1_/FVC at baseline [14% (95% CI: 8%, 20%) for men and 7% (95% CI: −10%, 21%) for women]. The probability of true regional differences was low.

**Conclusions:**

People with a higher FVC adjusted for age, sex, and height have a longer survival. Regional adjustments to lung function standards are inappropriate when assessing prognosis.

Key messages

- This research aimed to identify which spirometric measure [forced vital capacity (FVC) or forced expiratory volume in 1 second to FVC ratio (FEV_1_/FVC)] better predicts longevity and to test whether this is consistent across populations.
- We found that low FVC has a clearer association with mortality, with comparable results across populations.
- Our findings suggest that much of the mortality ascribed to chronic obstructive pulmonary disease in Asia and Africa is likely not due to airflow obstruction but rather due to low FVC.

## Introduction

Forced vital capacity (FVC) is a widely available spirometric measurement that is an approximation of the size of the lung (total lung capacity), and, in the USA, this is more closely related to survival than is the Forced Expiratory Volume in 1 second (FEV_1_) or FEV_1_ to FVC ratio (FEV_1_/FVC) [1–3]. The FVC is used clinically to identify restrictive lung disease but can also be used to assess the ‘severity’ of disease. The GOLD guidelines suggest the FEV_1_% predicted rather than the FEV_1_/FVC ratio to assess the severity of obstruction (https://GOLDCOPD.org), and we would argue that FEV_1_ is in effect acting as a surrogate for FVC or TLC, with which it is strongly correlated.

In the USA, it has been shown that although there are marked differences in ‘normal values’ between ‘White’ (European-American) and African-American populations, the prognostic significance of a given FVC is the same for a European-American and African-American individual of the same height, age, and sex [4–6]. There is, however, little information outside the USA on which to judge whether this equality of risk between populations is more generally the case.

Based on data from the Burden of Obstructive Lung Disease (BOLD) baseline survey, we have shown that ‘normal values’ of lung function can be divided into four relatively homogeneous world regions: ‘Europe’ (and high-income countries with predominant migration from Europe), ‘Near East’ (including Western and Central Asia and North Africa), ‘Sub-Saharan Africa’ (including the Caribbean), and ‘Far East’ (including South, South-East, and East Asia) [7]. Although the relation of FVC to age and height is different in each of these regions, it is not known whether this affects the relation of the FVC to survival.

Now, we have used data from the baseline and the follow-up of the BOLD cohort to test which baseline spirometric measurement, FVC or FEV_1_/FVC ratio, has the strongest association with longevity, and to test whether, after stratifying for sex and adjusting for age, smoking status, height, and weight, these show similar effects in different populations. The answers to these questions will determine whether universal standards are possible for spirometry and which those standards should be.

## Methods

### Study population

The data presented here are from the 2019–21 follow-up of individuals initially studied in selected sites from the BOLD study (2003–16) [8]. Full details are provided elsewhere [9]. The initial selection of sites for BOLD was based on the ability to undertake the study and was intended to cover all major world regions. In each site, a representative sample was selected of all those who were aged 40 years and over and were not institutionalized. Sites were selected for follow-up if they were in low- or middle-income countries [Benin, India (three sites), Jamaica, Kyrgyzstan (2 sites), Malawi, Morocco, Nigeria, Pakistan, the Philippines, Sudan, and Tunisia] or in the Nordic region (Iceland, Norway, Sweden, and Estonia). Those with complete spirometry data at the BOLD baseline visit were invited for follow-up. There was a single follow-up in each site.

### Mortality data

The endpoint was death from any cause other than accidents or violence. Mortality was based on questions asked to the household members and neighbours, including cause of death and time of death. Among the Nordic sites, the date of death was provided by the country’s death registry. If the date of death was not recorded, the date of death was estimated as halfway between the BOLD baseline visit and the follow-up when the death was reported.

### Lung function data

Lung function (FEV_1_ and FVC, and FEV_1_/FVC) was measured using an EasyOne spirometer (ndd Medizintechnik AG, Zurich, Switzerland), pre and postadministration of 200 mcg salbutamol through an inhalation chamber. All curves were centrally reviewed and scored based on ATS/ERS acceptability and repeatability criteria current at the time. As chronic obstructive pulmonary disease has generally been identified with obstruction that is not reversed by bronchodilators, we used postbronchodilator values in the analysis.

### Statistical analysis

Data were expressed as means and standard deviation (SD). Sex-specific univariable and multivariable Cox proportional hazard models were used to assess the association of FVC and FEV_1_/FVC ratio with mortality. The multivariable models were adjusted for age, smoking status, height, and weight. Inverse probability weighting was used to take account of variable follow-up of the sample [10, 11]. The results across all sites were summarized using random effects meta-analysis, and we used forest plots to explore the between-site variation in the association of FVC and FEV_1_/FVC with mortality.

To estimate absolute mortality rates at baseline (rather than just Hazard Ratios), we used a fully parametric Gompertz regression model. Mortality rates were adjusted for age and height. For illustration, we plotted mortality at age 55 years and at a typical height (170 cm for men and 160 cm for women). To illustrate the association, we drew graphs of mortality against FVC expressed as centiles for men and women separately. FVC centiles were estimated from *z*-scores in an earlier BOLD analysis of baseline lung function in relation to sex, age, and height-squared [7].

The statistical analysis was performed using Stata, version 17.

### Role of the funding source

The funders of the study had no role in study design, data collection, data analysis, data interpretation, or writing of the report.

## Results

**Figure 1:**
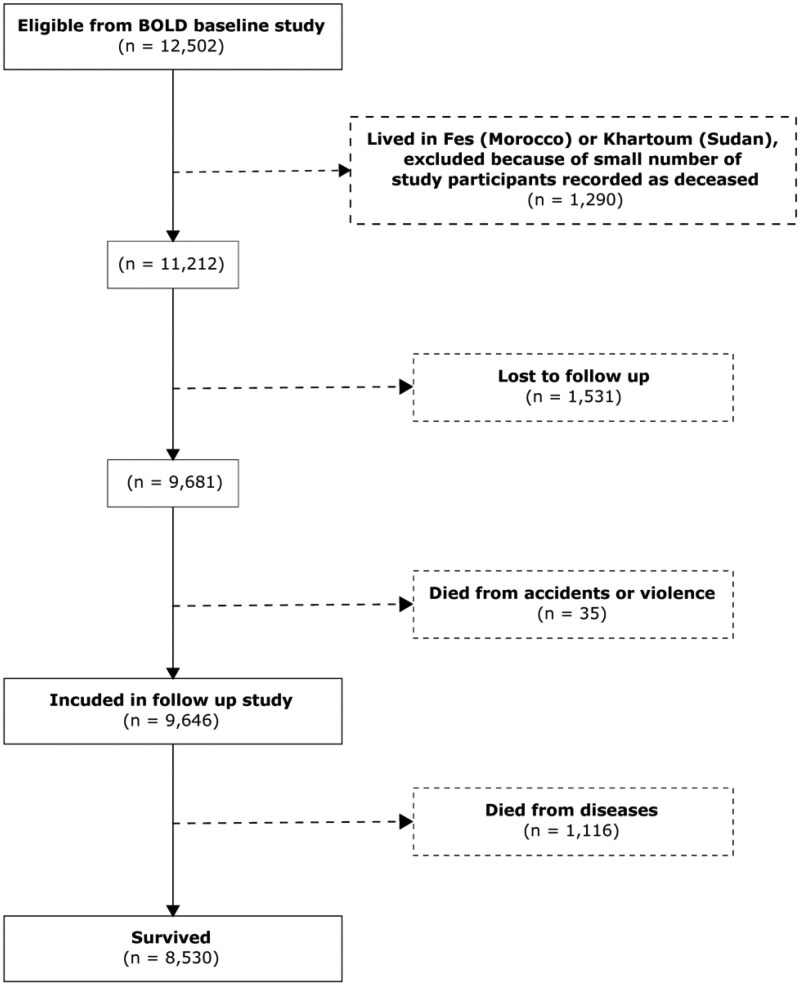
Flow diagram showing inclusion and exclusion from study.

Of 12 502 eligible individuals, 2540 (20.3%) were lost to follow-up, and 35 (0.3%) died as a result of accidents or violence (Fig. 1). These were excluded from all further analysis (Supplementary Table 1, in the supplementary material). Those lost to follow-up were slightly older and substantially more likely to be never smokers, but were otherwise similar at baseline to those who were followed up. Of the remaining 9927, 1120 (11.3%) had died from other causes (Table 1). Participants who were either unwilling to participate in the follow-up study or were known to be living away from their original address were included in the survival analysis if their vital status was known.

**Table 1:** Follow-up time and crude mortality for participants stratified by sex and Burden of Obstructive Lung Disease study site.

A. Men					
Site	Total included, *n* (%)	Survived, *n* (% of included)	**Died, *n* (% of included)** ^a^	Follow-up time, years, mean (SD)	Crude mortality rate per 100 000 individuals per year
Benin (Sèmè-Kpodji)	279 (91)	270 (97)	9 (3)	6.37 (0.58)	506
Estonia (Tartu)	313 (100)	199 (64)	114 (36)	9.21 (2.72)	3955
Iceland (Reykjavik)	364 (90)	265 (72)	99 (27)	12.93 (3.55)	2103
India (Kashmir)	76 (18)	66 (87)	10 (13)	9.48 (1.58)	1388
India (Mysore)	257 (99)	242 (94)	15 (6)	7.47 (2.94)	780
India (Pune)	496 (98)	425 (86)	71 (14)	10.37 (1.99)	1380
Jamaica	143 (57)	138 (96)	5 (3)	5.67 (0.73)	617
Kyrgyzstan (Chui)	257 (91)	241 (94)	16 (6)	6.21 (0.85)	1003
Kyrgyzstan (Naryn)	318 (96)	293 (92)	25 (8)	6.04 (0.86)	1302
Malawi (Chikwawa)	206 (90)	187 (91)	19 (9)	4.58 (0.89)	2014
Morocco (Fes)	112 (32)	112 (100)	0 (0)	10.70 (0.97)	n/a
Nigeria (Ife)	334 (97)	307 (92)	27 (8)	8.10 (1.17)	998
Norway (Bergen)	308 (94)	235 (76)	73 (23)	13.07 (3.90)	1,813
Pakistan (Karachi)	221 (81)	196 (88)	25 (11)	4.20 (0.81)	2,693
Philippines (Nampicuan-Talugtug)	354 (98)	290 (82)	64 (18)	9.84 (2.13)	1,837
Sudan (Khartoum)	42 (14)	40 (95)	2 (5)	7.72 (0.57)	617
Sweden (Uppsala)	263 (92)	237 (90)	26 (10)	12.33 (2.32)	802
Tunisia (Sousse)	229 (74)	202 (88)	27 (12)	10.14(1.44)	1,163
**Total**	**4572 (78)**	**3945 (86)**	**627 (14)**	**8.89 (3.46)**	**1543**
**Total** ^b^	**4418 (85)**	**3793 (86)**	**625 (14)**	**8.86 (3.50)**	**1597**

B. Women					
Site	Total included, *n* (%)	Survived, *n* (% of included)	**Died**, ***n* (% of included)**^a^	Follow-up time, years, mean (SD)	Crude mortality rate per 100 000 individuals per year
Benin (Sèmè-Kpodji)	363 (91)	358 (99)	5 (1)	6.45 (0.39)	214
Estonia (Tartu)	306 (100)	201 (66)	105 (34)	8.91 (2.81)	3851
Iceland (Reykjavik)	326 (92)	238 (73)	88 (27)	13.05 (3.28)	2068
India (Kashmir)	59 (17)	53 (90)	6 (10)	9.62 (1.45)	1057
India (Mysore)	346 (99)	339 (98)	7 (2)	7.49 (2.55)	270
India (Pune)	341 (99)	323 (95)	18 (5)	10.77 (1.48)	490
Jamaica	161 (47)	157 (98)	4 (2)	5.75 (0.52)	432
Kyrgyzstan (Chui)	581 (95)	561 (97)	20 (3)	6.26 (0.66)	550
Kyrgyzstan (Naryn)	529 (99)	509 (96)	20 (4)	6.14 (0.69)	616
Malawi (Chikwawa)	204 (94)	195 (96)	9 (4)	4.65 (0.79)	949
Morocco (Fes)	92 (22)	90 (98)	2 (2)	10.64 (1.19)	204
Nigeria (Ife)	525 (98)	500 (95)	25 (5)	8.23 (1.00)	579
Norway (Bergen)	314 (94)	248 (79)	66 (21)	13.68 (3.20)	1536
Pakistan (Karachi)	294 (85)	270 (92)	24 (8)	4.27 (0.76)	1912
Philippines (Nampicuan-Talugtug)	364 (99)	315 (88)	49 (13)	10.07 (1.87)	1337
Sudan (Khartoum)	35 (17)	35 (100)	0 (0)	7.87 (0.47)	n/a
Sweden (Uppsala)	245 (92)	219 (89)	26 (11)	12.42 (1.94)	854
Tunisia (Sousse)	270 (77)	251 (93)	19 (7)	10.37(0.99)	670
**Total**	**5355 (81)**	**4861 (91)**	**493 (9)**	**8.51 (3.26)**	**1082**
**Total** ^b^	**5228 (87)**	**4737 (91)**	**491 (9)**	**8.47 (3.28)**	**1109**

a Excluding deaths from accidents and violence.

b Excluding Morocco (Fes) and Sudan (Khartoum).

Table 2 gives the baseline information on the 4572 men and 5355 women included in the survival analysis. The mean ages of the men and women were 55 (SD 11) and 54 (SD 11) years, respectively. The mean weight was 73 (SD 17) kg in men and 67 (SD 16) kg in women. Mean height was 170 (SD 9) cm in men and 158 (SD 7) cm in women. Just over half (53%) of men and 17% of women had ever smoked, and 28% of men and 8% of women were current smokers at the time of the baseline survey. The mean FVC was 3.80 (SD 0.98) L in men and 2.70 (SD 0.69) L in women, and the mean FEV_1_ was 2.91 (SD 0.82) L in men and 2.14 (SD 0.58) L in women. The mean FEV_1_/FVC was 0.765 (SD 0.093) in men and 0.791 (SD 0.077) in women.

**Table 2:** Baseline characteristics of study participants included in the survival analysis (excluding those who could not be contacted and those who died of accidental deaths) stratified by sex and study site.

A. Men										
Study site	*N*	Age, mean (SD)	Weight, mean (SD)	Height, mean (SD)	Ever smoked (%)	Current smoking (%)	Pack years of smoking	**FEV_1_**, **mean (SD)**	FVC, mean (SD)	FEV_1_/FVC, mean (SD)
Benin (Sèmè-Kpodji)	279	52.9 (10.0)	71.7 (13.9)	170.7 (6.2)	14 (5.0)	12 (4.3)	0.5 (3.1)	2.60 (0.53)	3.30 (0.62)	0.787 (0.067)
Estonia (Tartu)	313	60.1 (11.7)	88.2 (16.6)	176.3 (7.1)	200 (63.9)	76 (24.3)	13.7 (17.2)	3.45 (0.82)	4.51 (0.90)	0.761 (0.082)
Iceland (Reykjavik)	364	56.0 (11.3)	91.7 (16.1)	179.4 (6.5)	257 (70.6)	75 (21.4)	14.8 (30.4)	3.56 (0.82)	4.67 (0.91)	0.758 (0.087)
India (Kashmir)	76	52.9 (11.1)	59.6 (9.3)	165.2 (6.3)	61 (80.3)	55 (72.3)	241.4 (295)	2.75 (0.83)	3.72 (0.70)	0.727 (0.121)
India (Mysore)	257	47.9 (8.2)	65.4 (9.5)	163.1 (5.7)	58 (22.6)	55 (21.4)	3.3 (8.7)	2.42 (0.69)	3.07 (0.75)	0.781 (0.085)
India (Pune)	496	53.2 (10.5)	59.8 (11.3)	163.8 (6.8)	102 (20.6)	78 (15.7)	1.1 (4.0)	2.46 (0.54)	3.11 (0.58)	0.789 (0.084)
Jamaica	143	57.5 (12.3)	69.9 (13.9)	171.2 (8.5)	91 (63.6)	47 (32.9)	26.1 (46.5)	2.77 (0.71)	3.58 (0.76)	0.770 (0.103)
Kyrgyzstan (Chui)	257	52.6 (9.2)	75.7 (14.6)	169.3 (6.9)	200 (77.8)	135 (52.5)	20.2 (22.8)	3.19 (0.74)	4.27 (0.77)	0.744 (0.097)
Kyrgyzstan (Naryn)	318	52.5 (10.1)	71.1 (12.4)	167.8 (6.3)	192 (60.4)	121 (38.1)	11.1 (15.3)	3.28 (0.65)	4.30 (0.73)	0.761 (0.084)
Malawi (Chikwawa)	206	55.0 (10.5)	57.2 (9.1)	166.2 (8.3)	98 (49.8)	64 (32.5)	4.1 (7.8)	2.65 (0.59)	3.55 (0.61)	0.746 (0.102)
Morocco (Fes)	112	54.8 (8.9)	74.2 (12.3)	168.8 (7.0)	70 (62.5)	24 (21.4)	16.2 (28.1)	3.15 (0.58)	3.99 (0.63)	0.787 (0.071)
Nigeria (Ife)	334	56.0 (12.3)	68.0 (13.3)	168.5 (6.7)	80 (24.0)	24 (7.2)	1.7 (7.2)	2.48 (0.62)	3.20 (0.66)	0.773 (0.093)
Norway (Bergen)	308	59.3 (12.6)	85.0 (14.6)	177.6 (7.2)	221 (71.8)	87 (28.3)	15.4 (17.2)	3.39 (0.84)	4.54 (0.94)	0.739 (0.095)
Pakistan (Karachi)	221	54.1 (9.3)	70.6 (16.0)	166.6 (8.1)	111 (50.5)	66 (30.0)	13.2 (29.7)	2.39 (0.65)	3.05 (0.72)	0.777 (0.104)
Philippines (Nampicuan-Talugtug)	354	53.7 (10.7)	57.9 (11.0)	164.3 (7.1)	272 (76.8)	182 (51.4)	21.4 (21.2)	2.46 (0.69)	3.22 (0.69)	0.755 (0.116)
Sudan (Khartoum)	42	52.9 (8.0)	75.3 (13.9)	171.4 (8.1)	26 (61.9)	15 (35.7)	11.3 (17.7)	2.67 (0.61)	3.40 (0.73)	0.783 (0.065)
Sweden (Uppsala)	263	58.5 (10.9)	86.4 (13.0)	178.0 (7.0)	181 (68.8)	35 (13.3)	12.4 (19.1)	3.53 (0.82)	4.67 (0.91)	0.754 (0.086)
Tunisia (Sousse)	229	53.2 (9.9)	78.4 (14.6)	170.1 (6.0)	180 (78.6)	120 (52.4)	28.8 (26.9)	3.10 (0.74)	3.98 (0.77)	0.774 (0.091)
**Total**	**4572**	**54.7 (11.1)**	**72.8 (17.4)**	**170.0 (8.7)**	**2414 (52.9)**	**1274 (27.9)**	**15.5 (52.6)**	**2.91 (0.82)**	**3.80 (0.98)**	**0.765 (0.093)**

B. Women										
Study site	*N*	Age, mean (SD)	Weight, mean (SD)	Height, mean (SD)	Ever smoked (%)	Current smoking (%)	Pack years of smoking	FEV_1_, mean (SD)	FVC, mean (SD)	FEV_1_/FVC, mean (SD)
Benin (Sèmè-Kpodji)	363	50.0 (9.2)	72.3 (16.1)	160.6 (6.2)	0 (0.0)	0 (0.0)	0.0 (0.0)	1.94 (0.41)	2.42 (0.46)	0.801 (0.069)
Estonia (Tartu)	306	60.8 (12.1)	75.0 (15.1)	162.0 (6.4)	96 (31.4)	39 (12.8)	3.5 (7.6)	2.50 (0.68)	3.17 (0.77)	0.784 (0.072)
Iceland (Reykjavik)	326	56.5 (11.7)	76.0 (15.6)	165.8 (6.3)	202 (62.0)	73 (22.4)	10.0 (13.5)	2.55 (0.62)	3.31 (0.67)	0.765 (0.087)
India (Kashmir)	59	53.2 (11.7)	54.3 (11.1)	153.3 (6.3)	22 (37.3)	17 (28.8)	94.1 (207)	1.92 (0.52)	2.53 (0.48)	0.755 (0.120)
India (Mysore)	346	45.9 (6.3)	59.7 (10.2)	155.2 (4.8)	5 (1.5)	4 (1.1)	0.1 (1.4)	1.79 (0.41)	2.22 (0.46)	0.806 (0.061)
India (Pune)	341	50.9 (8.5)	50.2 (10.0)	151.6 (6.1)	1 (0.3)	1 (0.3)	0.2 (2.8)	1.80 (0.37)	2.21 (0.40)	0.810 (0.072)
Jamaica	161	56.3 (11.5)	79.3 (18.6)	161.4 (6.5)	27 (16.8)	11 (6.8)	2.2 (9.3)	1.94 (0.48)	2.46 (0.52)	0.787 (0.094)
Kyrgyzstan (Chui)	581	52.5 (9.1)	72.9 (14.6)	157.3 (6.7)	44 (7.6)	27 (4.7)	0.9 (5.3)	2.36 (0.49)	3.00 (0.56)	0.788 (0.070)
Kyrgyzstan (Naryn)	529	53.0 (10.3)	68.0 (13.6)	155.4 (6.3)	13 (2.5)	2 (0.4)	0.1 (1.8)	2.36 (0.47)	2.98 (0.53)	0.791 (0.061)
Malawi (Chikwawa)	204	52.8 (10.4)	55.6 (11.9)	156.4 (7.2)	23 (11.6)	9 (4.5)	1.1 (4.9)	2.11 (0.42)	2.69 (0.48)	0.783 (0.072)
Morocco (Fes)	92	52.7 (9.3)	73.7 (12.5)	156.4 (6.4)	0 (0.0)	0 (0.0)	0.0 (0.0)	2.35 (0.50)	2.93 (0.59)	0.801 (0.063)
Nigeria (Ife)	525	54.8 (11.6)	66.6 (15.5)	159.0 (5.8)	20 (3.8)	1 (0.2)	0.1 (1.1)	1.87 (0.43)	2.34 (0.46)	0.795 (0.075)
Norway (Bergen)	314	60.4 (12.5)	70.8 (12.6)	164.4 (6.5)	183 (58.3)	87 (27.7)	10.2 (15.1)	2.48 (0.61)	3.27 (0.72)	0.755 (0.082)
Pakistan (Karachi)	294	49.2 (8.7)	65.7 (14.1)	154.3 (6.8)	26 (8.9)	16 (5.5)	2.8 (13.2)	1.76 (0.47)	2.15 (0.54)	0.822 (0.086)
Philippines (Nampicuan-Talugtug)	364	54.3 (10.4)	51.2 (10.9)	153.5 (6.0)	109 (30.0)	76 (20.9)	3.3 (7.4)	1.78 (0.47)	2.25 (0.51)	0.786 (0.094)
Sudan (Khartoum)	35	51.1 (10.9)	79.1 (17.8)	160.8 (7.4)	0 (0.0)	0 (0.0)	0.0 (0.0)	1.94 (0.47)	2.42 (0.55)	0.802 (0.062)
Sweden (Uppsala)	245	58.5 (10.7)	71.9 (14.4)	164.0 (6.2)	127 (51.8)	41 (16.7)	8.0 (12.1)	2.51 (0.60)	3.23 (0.67)	0.773 (0.075)
Tunisia (Sousse)	270	52.8 (8.8)	77.1 (13.4)	156.8 (6.7)	22 (8.2)	18 (6.7)	1.5 (6.9)	2.33 (0.58)	2.84 (0.63)	0.819 (0.054)
**Total**	**5355**	**53.7 (10.9)**	**67.3 (16.3)**	**158.1 (7.4)**	**920 (17.2)**	**422 (7.9)**	**3.5 (25.0)**	**2.14 (0.58)**	**2.70 (0.69)**	**0.791 (0.077)**

FVC, forced vital capacity; FEV_1_, 1-s forced expiratory volume; SD, standard deviation.

The proportion of participants followed up in the sites at Fes in Morocco (26%), Kashmir in India (19%), and Khartoum in Sudan (15%) was low due to the COVID-19 pandemic. Data from the sites in Morocco and Sudan were excluded from the survival analysis due to the small number of study participants recorded as dying (two in each site). The total number of study participants included in the survival analysis was 9927, of whom 1147 (10.5%) were reported dead at the follow-up. The mean (SD) follow-up time was 8.67 (3.30) years.

**Figure 2:**
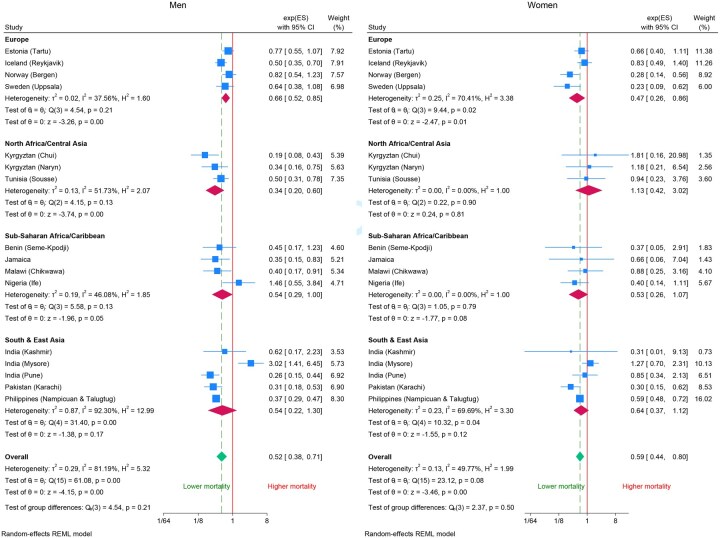
Forest plots of the association of forced vital capacity (FVC) with mortality by region and site (hazard ratios per 1 l increase in FVC adjusted for age, smoking status, height, and weight).

Figure 2 is a forest plot that shows the association between mortality and FVC as hazard ratios for men and women for each study site grouped by geographical region. There is considerable variation overall, but differences between regions are not significant (*P *= 0.21 for men and *P *= 0.50 for women). The hazard ratio associated with a 1 l increase in FVC is 0.52 [95% confidence interval (CI): 0.38, 0.71] for men and 0.59 (95% CI: 0.44, 0.80) for women.

**Figure 3:**
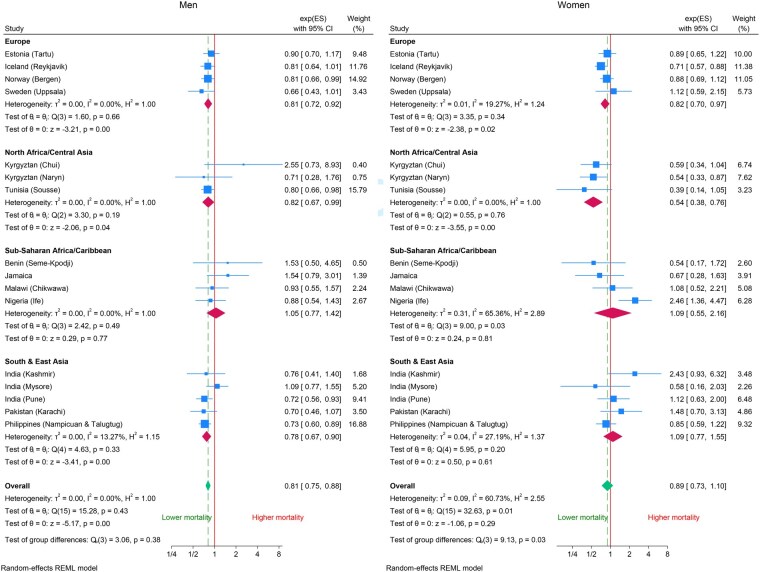
Forest plots of the association of forced expiratory volume in 1 second to forced vital capacity ratio (FEV_1_/FVC) with mortality by region and site (hazard ratios per 10% increase in the ratio adjusted for age, smoking status, height and weight).

Figure 3 shows the association of mortality with the FEV_1_/FVC ratio for men and women. There is little heterogeneity between regions among the men (*P *= 0.38), but there is heterogeneity among the women at both the site level and across regions (*P *= 0.03). The hazard ratio associated with a 10% increase in FEV_1_/FVC is 0.81 (95% CI: 0.75, 0.88) for men and 0.89 (95% CI: 0.73, 1.10) for women.

**Figure 4:**
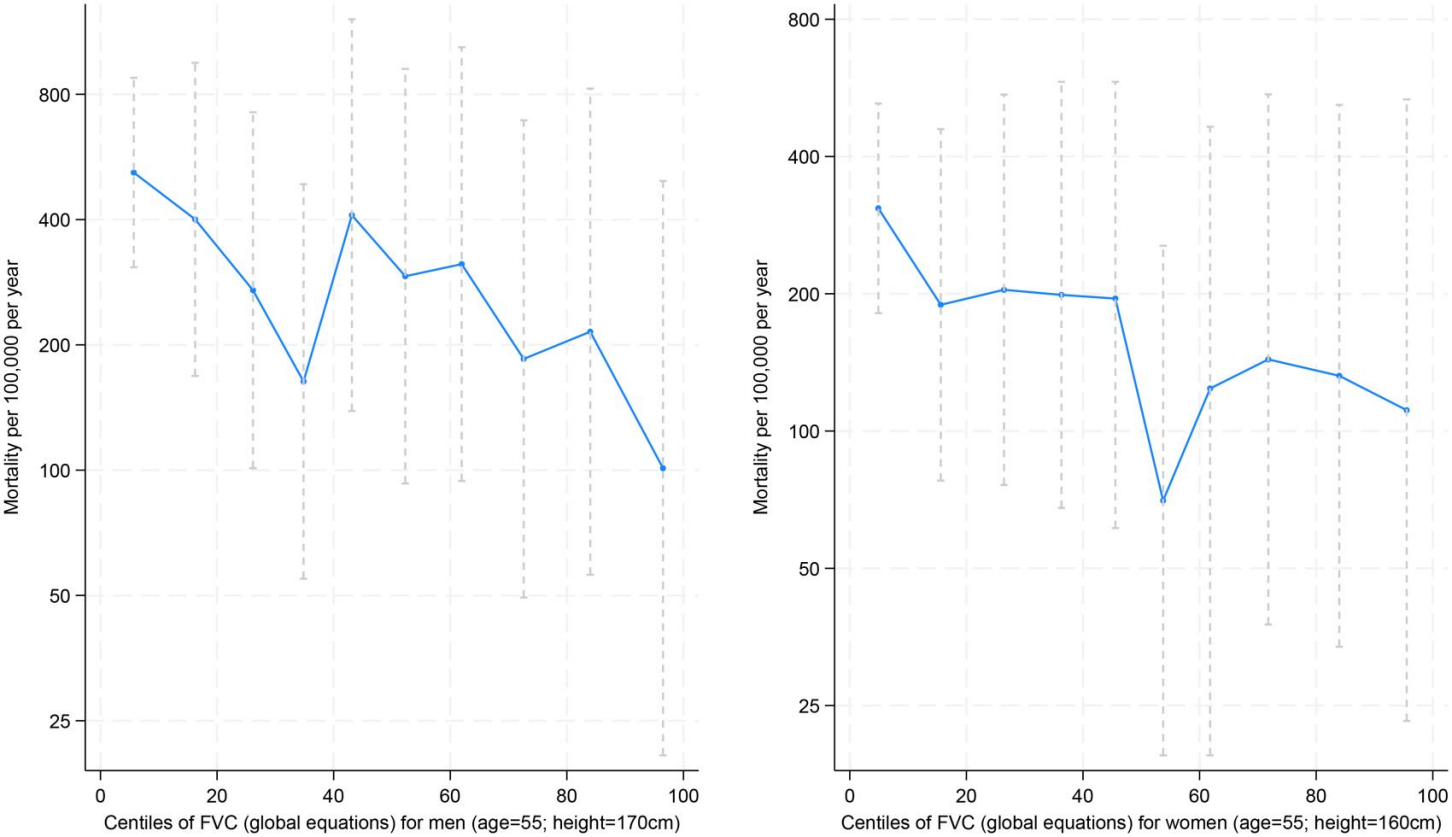
Distribution of forced vital capacity (FVC) (centiles) and its relationship with mortality using Burden of Obstructive Lung Disease (BOLD) study global reference equations (unadjusted for region). Centiles taken from the global values in Burney et al. [7]

Table 3 summarizes these associations per SD of lung function measurement and shows the independent effects of FVC and FEV_1_/FVC ratio. After adjusting for age, smoking status, height, and weight, and mutually adjusting for FVC and FEV_1_/FVC, the hazard ratios for men are 0.56 (95% CI: 0.42, 0.75) per SD of FVC and 0.86 (95% CI: 0.80, 0.92) per SD of FEV_1_/FVC ratio. The hazard ratios per SD for women were 0.72 (95% CI: 0.59, 0.89) for the FVC and 0.93 (0.79, 1.10) for the FEV_1_/FVC ratio. Figure 4 shows that in both sexes, mortality is lower where the FVC is higher.

**Table 3:** Association of spirometric measurements with mortality.

	Unadjusted		**Adjusted** ^a^		**Mutually adjusted** ^b^	
	HR	95% CI	Adj. HR	95% CI	Adj. HR	95% CI
**Men**						
FVC	0.37	(0.31, 0.45)	0.52	(0.38, 0.71)	0.56	(0.42, 0.75)
FEV_1_	0.42	(0.35, 0.49)	0.60	(0.47, 0.77)	–	–
FEV_1_/FVC	0.68	(0.58, 0.70)	0.82	(0.77, 0.89)	0.86	(0.80, 0.92)
**Women**						
FVC	0.39	(0.32, 0.48)	0.70	(0.57, 0.85)	0.72	(0.59, 0.89)
FEV_1_	0.41	(0.35, 0.50)	0.73	(0.64, 0.83)	–	–
FEV_1_/FVC	0.70	(0.61, 0.80)	0.92	(0.78, 1.08)	0.93	(0.79, 1.10)

Coefficients are per standard deviation to allow better comparison between lung function measurements.

FVC, forced vital capacity; FEV_1_, 1-s forced expiratory volume; HR: hazard ratio; Adj: adjusted; CI: confidence interval.

a Adjusted for age, smoking status, height and weight.

b Mutually adjusted for FVC, FEV_1_, age, smoking status, height, and weight.

## Discussion

This study has confirmed the association between FVC and all-cause mortality and that this association appears stronger than that between longevity and the FEV_1_/FVC ratio. The association of the FEV_1_/FVC ratio with mortality after adjusting for smoking was only significant among men. This weak association confirms earlier findings from the USA [2, 3].

It also shows that the association between FVC and mortality is constant across broad geographic regions. This is in spite of the wide heterogeneity in the association of FVC with age and height [11] and wide variations in mortality [12]. This is compatible with the conclusion of the American Thoracic Society statement, which noted that using ethnic adjustments to spirometric measurements is inappropriate when assessing prognosis [13]. It extends the evidence for this conclusion to include a wide variety of populations outside the USA. We did not look at ethnicity specifically, as most study sites were ethnically homogeneous, and ethnicity is hard to define. However, if ethnicity was important, we would have seen a regional difference in our results, and we did not.

We used random-effects meta-analysis to combine results. This gives broader confidence intervals than fixed-effects analysis, but is justified by the results that show substantial variation in results between sites.

The BOLD study has many strengths when answering the question of whether a single standard can be used to assess spirometric values. It uses a standardized protocol across all study sites. It covers a wide geographical area with a known variation in mean FVC and in the relation of the FVC to age and height [11]. The spirometry was quality controlled both by training all of the technicians involved in the study and by over-reading the spirometry centrally and asking for technicians whose quality started to fail to be retrained before continuing. This level of quality assurance has been shown to be essential in studies of lung function [14, 15]. The overall follow-up rate was 79.4%, and 11/18 (61%) sites had a >90% retention.

In low-income countries, mortality was estimated using information from relatives and neighbours. Other than excluding deaths from accidents and violence, we did not attempt to assess the cause of death. The fact of death, if reported, was regarded as reliable.

The COVID-19 pandemic made serious difficulties in some sites, and some of them did not complete the follow-up study. This may have weakened the power of the study, but we do not believe that it had any material influence that might have altered the conclusions from the analysis. Those lost to follow-up were slightly older and substantially more likely to be never smokers [13] but were otherwise similar at baseline to those who were followed up. We used inverse probability weighting to reduce any bias due to differential follow-up. Participants who were unable to provide adequate spirometry at baseline were excluded from the follow-up. It has been shown that these people have a worse prognosis, but their exclusion is unavoidable and unlikely to have altered the conclusions from our analysis. Although there was reasonable representation of the four broad geographical regions identified in the BOLD baseline survey, there are large areas that are not represented in the follow-up, most prominently China and the affluent countries of South-East and East Asia.

Our analysis used the Cox proportional hazards method. This method allows a parametric analysis of multiple variables simultaneously but assumes that the hazards are proportional. We tested this assumption in all the sites using Schoenfeld residuals and only two sites, Reykjavik and Mysore, infringed this assumption in the model for FVC in men, and only two sites, Reykjavik and Ife, among women. When these sites were removed from the analysis, there was very little change in the results (Supplementary Table 2, in the supplementary material).

We have focused our analysis on two main spirometric measurements, the FVC, a measurement of lung size, and the FEV_1_/FVC ratio, which is a measure of airflow adjusted for lung size. It has been common for the FEV_1_ alone to be used as a marker of ventilatory inadequacy [16, 17], and it has been suggested that this is a more reliable marker of outcome [18, 19]. Fletcher et al. used the FEV_1_ by itself because they suspected early on that their FVC measurements were unreliable. This is a common problem, as the FVC is a more difficult measurement. The FEV_1_ is, besides this, highly correlated with the FVC, and it is not entirely irrational to use this as a proxy for the FVC or the TLC. The main problem with this practice is that it leads to the erroneous impression that it is airflow obstruction that is driving mortality. Here, we show that it is the FVC (lung size) and not the FEV_1_/FVC (airflow adjusted for lung size) that is more strongly associated with mortality. This confirms the conclusions from earlier analyses of the Cardiovascular Health Study [2] and the Atherosclerosis Risk in Communities Study [3].

Those who have a low FVC have a similar prevalence of dyspnoea as do those with a low FEV_1_/FVC ratio [20], and cases with a low FVC, in the absence of spirometry, are likely to be misdiagnosed as having airflow obstruction, particularly in the countries of South, Southeast and East Asia, sub-Saharan Africa, and the Caribbean, where a low FVC is common [11]. The proximal and distal causes of death in these people are unclear, but it is unlikely to be respiratory failure. A low FVC is strongly associated with both diabetes and cardiovascular co-morbidities in all regions of the world [21] and is associated with central arterial stiffness [22, 23]. The name ‘small lung syndrome’ has been suggested to emphasize the close association between these three conditions [24]. The causes of the close association between these conditions are likely to arise in early life from shared risk factors [25], including low birth weight [26].

The analyses summarized in Fig. 4 show how FVC is related to mortality and suggest that a continuous risk could be provided based on outcomes. Moving from the use of arbitrary cut-offs, such as the lower limit of normal, to a continuous score of the FVC calibrated against outcomes, such as mortality, would provide a more rational basis for assessing prognosis than the use of ‘normal values’ on their own. The alternative is to define decision limits by estimating the values associated with particular outcomes or the relative benefits of intervention at different values [27]. This method has been used, for instance, to set limits for blood pressure in men and women [28]. The use of mortality as the outcome of choice has the advantage that it is clearly associated with the FVC, it has a clear definition, and is self-evidently of value to the individual. Establishing more precise risk scores will require larger cohorts of samples with good-quality spirometry and mortality data.

The use of regional adjustments for lung function is inappropriate when assessing survival but may be appropriate when making a diagnosis. The low FVC found very generally in Asia and Africa is part of what the CIBA symposium defined as ‘non-specific lung disease’ [29]. When considering the possibility of a specific restrictive lung disease such as sarcoidosis or silicosis, it would be appropriate to take account of the lower levels of vital capacity seen in the local population. The same dilemma does not occur when dealing with obstructive disease, as the FEV_1_/FVC ratio does not vary from population to population in the same way [7, 30].

In conclusion, our findings emphasize the importance of a low FVC in determining high mortality rates in the Far East, Sub-Saharan Africa, and the Caribbean. They also support the recent American Thoracic Society Statement that adjustment of spirometric findings for ethnicity is inappropriate, at least when assessing prognosis [15]. It also extends the evidence for this advice beyond the USA to most of the world, including sub-Saharan Africa and the Far East. It further demonstrates how survival analysis can, in principle, provide interpretation of lung function that is linked to patient outcomes.

## Ethics approval

All sites received approval from their local ethics committee, and the protocol was also approved by Imperial College London Research Ethics Committee (ref. 17IC4272). All participants provided informed consent.

## Supplementary Material

dyag093_Supplementary_Data

## Data Availability

De-identified participant data and questionnaires may be shared, after publication, on a collaborative basis upon reasonable request made to Dr Amaral (a.amaral@imperial.ac.uk). Requesting researchers will be required to submit an analysis plan.
