# Supplementary material for: A cohort study of forced vital capacity, airway obstruction, and survival in the multinational Burden of Obstructive Lung Disease study
Source: Int J Epidemiol. 2026 Jun 7;55(3):dyag093. doi: 10.1093/ije/dyag093 (PMC13242796; doi:10.1093/ije/dyag093)
Supplement: dyag093_Supplementary_Data [file dyag093_supplementary_data.zip › 01-Jun-2026_100903_ije-2026-01-0009-File007.docx]

**Supplementary Tables**

**TABLE S1: Inclusion in study**

| **Site** | **Eligible** | **Lost to follow-up**  **n (%)** | **Died from accidents and violence**  **n (%)** | **Total included**  **n (%)** |
| --- | --- | --- | --- | --- |
| Benin (Sèmè-Kpodji) | 706 | 64 (9.1) | 0 (0.0) | 642 (90.9) |
| Estonia (Tartu) | 619 | 0 (0.0) | 0 (0.0) | 619 (100) |
| Iceland (Reykjavik) | 757 | 67 (8.9) | 0 (0.0) | 690 (91.1) |
| India (Kashmir) | 771 | 634 (82.2) | 2 (0.3) | 135 (17.5) |
| India (Mysore) | 607 | 3 (0.5) | 1 (0.2) | 603 (99.3) |
| India (Pune) | 851 | 0 (0.0) | 14 (1.7) | 837 (98.4) |
| Jamaica | 594 | 289 (48.7) | 1 (0.2) | 304 (51.2) |
| Kyrgyzstan (Chui) | 894 | 50 (5.6) | 6 (0.7) | 838 (93.7) |
| Kyrgyzstan (Naryn) | 865 | 16 (1.9) | 2 (0.2) | 847 (97.9) |
| Malawi (Chikwawa) | 448 | 38 (8.5) | 0 (0.0) | 410 (91.5) |
| Morocco (Fes) | 770 | 566 (73.5) | 0 (0.0) | 204 (26.5) |
| Nigeria (Ife) | 884 | 21 (2.4) | 4 (0.5) | 859 (97.2) |
| Norway (Bergen) | 661 | 39 (5.9) | 0 (0.0) | 622 (94.1) |
| Pakistan (Karachi) | 616 | 99 (16.1) | 2 (0.3) | 515 (83.6) |
| Philippines (Nampicuan-Talugtug) | 727 | 6 (0.8) | 3 (0.4) | 718 (98.8) |
| Sudan (Khartoum) | 520 | 443 (85.2) | 0 (0.0) | 77 (14.8) |
| Sweden (Uppsala) | 551 | 43 (7.8) | 0 (0.0) | 508 (92.2) |
| Tunisia (Sousse) | 661 | 162 (24.5) | 0 (0.0) | 499 (75.5) |
| **Total** | **12,502** | **2,540 (20.3)** | **35 (0.3)** | **9,927 (79.4)** |
| **Total*** | **11,212** | **1,531 (13.7)** | **35 (0.3)** | **9,646 (86.0)** |

*Excluding Morocco (Fes) and Sudan (Khartoum).

**Table S2 Association of spirometric measurements with mortality. Coefficients are per standard deviation to allow better comparison between lung function measurements.**

|  | | **Adjusted** | | | | **After removal of sites violating proportional hazards assumptions** | | | |
| --- | --- | --- | --- | --- | --- | --- | --- | --- | --- |
|  | |  | | | |  | | | |
|  | | **Adj. HR** | | **95% CI** | | **Adj. HR** | | **95% CI** | |
| **MEN** | |  | |  | |  | |  | |
| FVC | | 0.52 | | (0.38, 0.71) | | 0.47 | | (0.36, 0.61) | |
| FEV_1_ | | 0.60 | | (0.47, 0.77) | | 0.61 | | (0.46, 0.80) | |
| FEV_1_/FVC | | 0.82 | | (0.77, 0.89) | | 0.81 | | (0.75, 0.88) | |
|  | |  | |  | |  | |  | |
| **WOMEN** | |  | |  | |  | |  | |
| FVC | | 0.70 | | (0.57, 0.85) | | 0.69 | | (0.54, 0.87) | |
| FEV_1_ | | 0.73 | | (0.64, 0.83) | | 0.72 | | (0.61, 0.84) | |
| FEV_1_/FVC | | 0.92 | | (0.78, 1.08) | | 0.89 | | (0.80, 1.00) | |
|  |  | |  | |  | |  | |  |

Adj, adjusted; CI, Confidence Interval; FEV_1_, one-second Forced Expiratory Volume; FVC, Forced Vital Capacity; HR: Hazard Ratio.
