# Supplementary material for: A cohort study of forced vital capacity, airway obstruction, and survival in the multinational Burden of Obstructive Lung Disease study
Source: Int J Epidemiol. 2026 Jun 7;55(3):dyag093. doi: 10.1093/ije/dyag093 (PMC13242796; doi:10.1093/ije/dyag093)
Supplement: dyag093_Supplementary_Data [file dyag093_supplementary_data.zip › 04-Jun-2026_055306_ije-2026-01-0009-File007.docx]

**Supplementary Tables**

**TABLE S1: Inclusion in study**

| **Site** | **Eligible** | **Lost to follow-up**  **n (%)** | **Died from accidents and violence**  **n (%)** | **Total included**  **n (%)** |
| --- | --- | --- | --- | --- |
| Benin (Sèmè-Kpodji) | 706 | 64 (9.1) | 0 (0.0) | 642 (90.9) |
| Estonia (Tartu) | 619 | 0 (0.0) | 0 (0.0) | 619 (100) |
| Iceland (Reykjavik) | 757 | 67 (8.9) | 0 (0.0) | 690 (91.1) |
| India (Kashmir) | 771 | 634 (82.2) | 2 (0.3) | 135 (17.5) |
| India (Mysore) | 607 | 3 (0.5) | 1 (0.2) | 603 (99.3) |
| India (Pune) | 851 | 0 (0.0) | 14 (1.7) | 837 (98.4) |
| Jamaica | 594 | 289 (48.7) | 1 (0.2) | 304 (51.2) |
| Kyrgyzstan (Chui) | 894 | 50 (5.6) | 6 (0.7) | 838 (93.7) |
| Kyrgyzstan (Naryn) | 865 | 16 (1.9) | 2 (0.2) | 847 (97.9) |
| Malawi (Chikwawa) | 448 | 38 (8.5) | 0 (0.0) | 410 (91.5) |
| Morocco (Fes) | 770 | 566 (73.5) | 0 (0.0) | 204 (26.5) |
| Nigeria (Ife) | 884 | 21 (2.4) | 4 (0.5) | 859 (97.2) |
| Norway (Bergen) | 661 | 39 (5.9) | 0 (0.0) | 622 (94.1) |
| Pakistan (Karachi) | 616 | 99 (16.1) | 2 (0.3) | 515 (83.6) |
| Philippines (Nampicuan-Talugtug) | 727 | 6 (0.8) | 3 (0.4) | 718 (98.8) |
| Sudan (Khartoum) | 520 | 443 (85.2) | 0 (0.0) | 77 (14.8) |
| Sweden (Uppsala) | 551 | 43 (7.8) | 0 (0.0) | 508 (92.2) |
| Tunisia (Sousse) | 661 | 162 (24.5) | 0 (0.0) | 499 (75.5) |
| **Total** | **12,502** | **2,540 (20.3)** | **35 (0.3)** | **9,927 (79.4)** |
| **Total*** | **11,212** | **1,531 (13.7)** | **35 (0.3)** | **9,646 (86.0)** |

*Excluding Morocco (Fes) and Sudan (Khartoum).

**Table S2 Association of spirometric measurements with mortality. Coefficients are per standard deviation to allow better comparison between lung function measurements.**

|  | | **Adjusted** | | | | **After removal of sites violating proportional hazards assumptions** | | | |
| --- | --- | --- | --- | --- | --- | --- | --- | --- | --- |
|  | |  | | | |  | | | |
|  | | **Adj. HR** | | **95% CI** | | **Adj. HR** | | **95% CI** | |
| **MEN** | |  | |  | |  | |  | |
| FVC | | 0.52 | | (0.38, 0.71) | | 0.47 | | (0.36, 0.61) | |
| FEV_1_ | | 0.60 | | (0.47, 0.77) | | 0.61 | | (0.46, 0.80) | |
| FEV_1_/FVC | | 0.82 | | (0.77, 0.89) | | 0.81 | | (0.75, 0.88) | |
|  | |  | |  | |  | |  | |
| **WOMEN** | |  | |  | |  | |  | |
| FVC | | 0.70 | | (0.57, 0.85) | | 0.69 | | (0.54, 0.87) | |
| FEV_1_ | | 0.73 | | (0.64, 0.83) | | 0.72 | | (0.61, 0.84) | |
| FEV_1_/FVC | | 0.92 | | (0.78, 1.08) | | 0.89 | | (0.80, 1.00) | |
|  |  | |  | |  | |  | |  |

Adj, adjusted; CI, Confidence Interval; FEV_1_, one-second Forced Expiratory Volume; FVC, Forced Vital Capacity; HR: Hazard Ratio.

**BOLD (Burden of Obstructive Lung Disease) Collaborative Research Group members**

**Albania**: Hasan Hafizi (principal investigator [PI]), Anila Aliko, Donika Bardhi, Holta Tafa, Natasha Thanasi, Arian Mezini, Alma Teferici, Dafina Todri, Jolanda Nikolla, and Rezarta Kazasi (Tirana University Hospital Shefqet Ndroqi, Albania); **Algeria**: Hamid Hacene Cherkaski (PI), Amira Bengrait, Tabarek Haddad, Ibtissem Zgaoula, Maamar Ghit, Abdelhamid Roubhia, Soumaya Boudra, Feryal Atoui, Randa Yakoubi, Rachid Benali (Department of Pneumology, Faculty of Medicine, Annaba, Algeria), Abdelghani Bencheikh and Nadia Ait-Khaled (Department of Epidemiology and Prevention, EPHS ElHadjar, Algeria); **Australia**: Christine Jenkins (PI), Guy Marks (PI), Tessa Bird, Paola Espinel, Kate Hardaker, Brett Toelle (Woolcock Institute of Medical Research, Sidney, Australia); **Austria**: Michael Studnicka (PI), Torkil Dawes, Bernd Lamprecht, and Lea Schirhofer (Department of Pulmonary Medicine, Paracelsus Medical University, Salzburg, Austria); **Benin**: Herve Lawin (PI), Arsene Kpangon, Karl Kpossou, Gildas Agodokpessi, Paul Ayelo, Benjamin Fayomi, Rolus Atrokpo, Gaston Hounton, Dieudonnè Yadjodo (Unit of Teaching and Research in Occupational and Environmental Health, University of Abomey Calavi, Cotonou, Benin); **Cameroon**: Bertrand Mbatchou (PI), Atongno Humphrey Ashu (Douala General Hospital, Douala, Cameroon); **Canada**: Wan C Tan (PI) and Wen Wang (iCapture Center for Cardiovascular and Pulmonary Research, University of British Columbia, Vancouver, BC, Canada); **China**: NanShan Zhong (Principal Investigator [PI]), Shengming Liu, Jiachun Lu, Pixin Ran, Dali Wang, Jin-ping Zheng, and Yumin Zhou (Guangzhou Institute of Respiratory Health, First Affiliated Hospital of Guangzhou Medical College, Guangzhou, China); **Estonia**: Rain Jõgi (PI), Hendrik Laja, Katrin Ulst, Vappu Zobel, Toomas-Julius Lill, Katrin Kiili, and Ira Laanelepp (Lung Clinic, Tartu University Hospital, Tartu, Estonia); **Germany**: Tobias Welte (PI), Isabelle Bodemann, Henning Geldmacher, and Alexandra Schweda-Linow (Dept of Pneumology, Hannover Medical School and German Center of Lung Research, Hannover, Germany); **Iceland**: Thorarinn Gislason (PI), Bryndis Benedikdtsdottir, Kristin Jörundsdottir, Lovisa Gudmundsdottir, Sigrun Gudmundsdottir, Gunnar Gudmundsson, Elin Helga Thorarinsdottir, and Hjördis Sigrun Pálsdottir (Department of Allergy, Respiratory Medicine, and Sleep, Landspitali University Hospital, Reykjavik, Iceland); **India**: Mahesh Padukudru Anand (PI) (JSS Medical College, JSSAHER, Mysuru, India); Parvaiz A Koul (PI), Sajjad Malik, Nissar A Hakim, and Umar Hafiz Khan (Sher-i-Kashmir Institute of Medical Sciences, Srinagar, J&K, India); Rohini Chowgule (PI), Vasant Shetye, Jonelle Raphael, Rosel Almeda, Mahesh Tawde, Rafiq Tadvi, Sunil Katkar, Milind Kadam, Rupesh Dhanawade, and Umesh Ghurup (Indian Institute of Environmental Medicine, Mumbai, India); Sanjay Juvekar (PI), Siddhi Hirve, Somnath Sambhudas, Bharat Chaidhary, Meera Tambe, Savita Pingale, Arati Umap, Archana Umap, Nitin Shelar, Sampada Devchakke, Sharda Chaudhary, Suvarna Bondre, Savita Walke, Ashleshsa Gawhane, Anil Sapkal, Rupali Argade, Vijay Gaikwad, Dhiraj Agrawal, Babu Pawar, Shalan Mhetre, Namdev Kale, and Shirish Kathale (Vadu Rural Health Program, Pune, India); Sundeep Salvi (PI), Bill Brashier, Jyoti Londhe, and Sapna Madas (Chest Research Foundation, Pune, India); **Jamaica**: Althea Aquart-Stewart (PI), Akosua Francia Aikman (University of the West Indies, Kingston, Jamaica); **Kyrgyzstan**: Talant M Sooronbaev (PI), Bermet M Estebesova, Meerim Akmatalieva, Saadat Usenbaeva, Jypara Kydyrova, Eliza Bostonova, Ulan Sheraliev, Nuridin Marajapov, Nurgul Toktogulova, Berik Emilov, Toktogul Azilova, Gulnara Beishekeeva, Nasyikat Dononbaeva, and AijamalTabyshova (Pulmunology and Allergology Department, National Centre of Cardiology and Internal Medicine, Bishkek, Kyrgyzstan); **Malawi**: Kevin Mortimer (Baseline PI), Wezzie Nyapigoti, Ernest Mwangoka, Mayamiko Kambwili, Martha Chipeta, Gloria Banda, Suzgo Mkandawire, Justice Banda, Graham Devereux (Follow-up PI), Jamie Rylance, Martin Njoroge, Catherine Chirwa, Chifundo Mhango, Edgar Ngwira, Faith Zumazuma, Frank Jonas, and Patrick Mjojo (the Malawi Liverpool Wellcome Trust, Blantyre, Malawi); **Malaysia**: Li-Cher Loh (PI), Abdul Rashid, and Siti Sholehah (Royal College of Surgeons in Ireland and University College Dublin Malaysia Campus (RUMC)); **Morocco**: Mohamed C Benjelloun (Baseline PI), Chakib Nejjari, Mohamed Elbiaze, Karima El Rhazi (Follow-up PI), Manelle Rjimati, Btissame ElHarche, Reda Benjelloun, and Yassin Chefchaou (Laboratoire d’épidémiologie, Recherche Clinique et Santé Communautaire, Fès, Morroco); **The Netherlands**: E F M Wouters and G J Wesseling (Maastricht University Medical Center, Maastricht, the Netherlands); **Nigeria**: Daniel Obaseki (PI), Gregory Erhabor, Olayemi Awopeju, and Olufemi Adewole (Obafemi Awolowo University, Ile-Ife, Nigeria); **Norway**: Amund Gulsvik (Baseline PI), Tina Endresen, Lene Svendsen (Department of Thoracic Medicine, Institute of Medicine, University of Bergen, Bergen, Norway), and Rune Nielsen (Follow-up PI), Marit Aardal, Hildegunn B Fleten, Gerd Eli Dale, Eli Nordeide, Malin P Grøttveit, Åsa Skjelde, Ane Aamli Gagnat, Anders Ørskov Rotevatn, Marta Erdal (Department of Clinical Science, University of Bergen, Bergen, Norway); **Pakistan**: Asaad A Nafees (PI), Muhammad Irfan, Hasan Nawaz Tahir, Muhammad Noman, Roman Ul Haq (Aga Khan Univeristy, Karachi, Pakistan); **Philippines**: Luisito F Idolor (Baseline PI), Teresita S de Guia, Norberto A Francisco, Camilo C Roa, Fernando G Ayuyao, Cecil Z Tady, Daniel T Tan, Sylvia Banal-Yang, Vincent M Balanag, Jr, Maria Teresita N Reyes, Renato B Dantes, and Stefanni Nonna M Paraguas (Follow-up PI) (Lung Centre of the Philippines and Philippine Heart Centre, Philippine General Hospital, Nampicuan and Talugtug, the Philippines); Renato B Dantes (Baseline PI), Lourdes Amarillo, Lakan U Berratio, Lenora C Fernandez, Norberto A Francisco, Gerard S Garcia, Teresita S de Guia, Luisito F Idolor, Sullian S Naval, Thessa Reyes, Camilo C Roa, Jr, Ma Flordeliza Sanchez, and Leander P Simpao (Philippine College of Chest Physicians, Manila, the Philippines); **Poland**: Ewa Nizankowska-Mogilnicka (PI), Jakub Frey, Rafal Harat, Filip Mejza, Pawel Nastalek, Andrzej Pajak, Wojciech Skucha, Andrzej Szczeklik, and Magda Twardowska, (Division of Pulmonary Diseases, Department of Medicine, Jagiellonian University School of Medicine, Krakow, Poland); **Portugal**: Cristina Bárbara (PI), Fátima Rodrigues, Hermínia Dias, João Cardoso, João Almeida, Maria João Matos, Paula Simão, Moutinho Santos, and Reis Ferreira (the Portuguese Society of Pneumology, Lisbon, Portugal); **Saudi Arabia**: M Al Ghobain (PI), H Alorainy (PI), E El-Hamad, M Al Hajjaj, A Hashi, R Dela, R Fanuncio, E Doloriel, I Marciano, and L Safia (Saudi Thoracic Society, Riyadh, Saudi Arabia); **South Africa**: Eric Bateman (Baseline PI), Anamika Jithoo (Baseline PI), Desiree Adams, Edward Barnes, Jasper Freeman, Anton Hayes, Sipho Hlengwa, Christine Johannisen, Mariana Koopman, Innocentia Louw, Ina Ludick, Alta Olckers, Johanna Ryck, Janita Storbeck, and Richard van Zyl-Smit (Follow-up PI) (University of Cape Town Lung Institute, Cape Town, South Africa); **Sri Lanka**: Kirthi Gunasekera (PI), Rajitha Wickremasinghe (Medical Research Institute, Central Chest Clinic, Colombo, Sri Lanka); **Sudan**: Asma Elsony (Baseline PI), Hana A Elsadig, Nada Bakery Osman, Bandar Salah Noory, Monjda Awad Mohamed, Hasab Alrasoul Akasha Ahmed Osman, Namarig Moham ed Elhassan, Abdel Mu‘is El Zain, Marwa Mohamed Mohamaden, Suhaiba Khalifa, Mahmoud Elhadi, Mohand Hassan, Dalia Abdelmonam, Rana Ahmed (Follow-up PI), Rashid Osman, Hind Eltigani, Najlaa Mohamed Abass, Ahmed Beriar Ahmed, Sahar AlaElddin (Epidemiological Laboratory, Khartoum, Sudan); **Sweden**: Christer Janson (PI), Inga Sif Olafsdottir, Katarina Nisser, Ulrike Spetz-Nyström, Gunilla Hägg, Gun-Marie Lund, Andrei Malinovschi, Eva Wallberg, Birgitta Appelfeldt, and Mona Andrén (Department of Medical Sciences: Respiratory Medicine and Allergology, Uppsala University, Uppsala, Sweden); **Trinidad and Tobago**: Terence Seemungal (PI), Fallon Lutchmansingh, Liane Conyette (University of the West Indies, St Augustine, Trinidad and Tobago); **Tunisia**: Imed Harrabi (Baseline PI), Myriam Denguezli (Follow-up PI), Zouhair Tabka (deceased), Hager Daldoul, Zaki Boukheroufa, Firas Chouikha, Wahbi Belhaj Khalifa, Safa Hsan, Nadia Lakhdar, and Mounir Landolsi (University Hospital Farhat Hached, Faculté de Médecine, Sousse, Tunisia); **Turkey**: Ali Kocabaş (PI), Attila Hancioglu, Ismail Hanta, Sedat Kuleci, Ahmet Sinan Turkyilmaz, Sema Umut, and Turgay Unalan (Department of Chest Diseases, Cukurova University School of Medicine, Adana, Turkey); **UK**: Peter G J Burney (Baseline and Follow-up PI), Anamika Jithoo, Louisa Gnatiuc, Hadia Azar, Jaymini Patel, Caron Amor, James Potts, Michael Tumilty, Fiona McLean, Risha Dudhaiya, Andre F S Amaral (Project lead), Octavia Mulhern, Emmanouil Bagkeris, Jasleen Gegic, Paul Cullinan, Cosetta Minelli (National Heart and Lung Institute, Imperial College London, London, UK); **USA**: A Sonia Buist (Baseline PI) (Oregon Health & Science University, Portland, OR), Mary Ann McBurnie, William M Vollmer, Suzanne Gillespie (Kaiser Permanente Center for Health Research, Portland, OR); Sean Sullivan (University of Washington, Seattle, WA); Todd A Lee, Kevin B Weiss, (Northwestern University, Chicago, IL); Robert L Jensen, Robert Crapo (Latter Day Saints Hospital, Salt Lake City, Utah); Paul Enright (University of Arizona, Tucson, AZ); David M Mannino (PI), John Cain, Rebecca Copeland, Dana Hazen, and Jennifer Methvin, (University of Kentucky, Lexington, KY); Vanessa Garcia Larsen (John Hopkins Bloomberg School of Public Health, Baltimore, MD)
